# Timely course of SARS-CoV-2 infections and vaccinations in patients with hemato-oncological diseases: analysis of a real-life cohort

**DOI:** 10.1016/j.esmoop.2023.101559

**Published:** 2023-04-25

**Authors:** M.J. Mair, M. Mitterer, T. Buratti, L. Berchtold, D. Fong, M. Preusser

**Affiliations:** 1 Division of Oncology, Department of Medicine I, Medical University of Vienna, Vienna, Austria; 2 Hemato-Oncological Day Hospital Unit, Franz Tappeiner Hospital, Meran/Merano, Italy

**Keywords:** SARS-CoV-2, COVID-19, cancer, pandemic, vaccination

## Abstract

**Background:**

The severe acute respiratory syndrome coronavirus 2 (SARS-CoV-2) pandemic has particularly impacted patients with hemato-oncological malignancies, as they showed not only a higher propensity for severe courses but also weaker immune responses after vaccination. Still, data on the influence of pandemic waves and vaccinations on outcomes are rare. This study aimed to analyze the timely course of infections and vaccinations in a real-life cohort of patients with hemato-oncological diseases.

**Methods:**

In this cohort study, 1817 patients with hemato-oncological diseases from 1 February 2020 to 15 December 2022 at the ‘Franz Tappeiner’ Hospital in Merano/Meran, Italy, were followed for SARS-CoV-2 infections and vaccinations.

**Results:**

Of 1817 patients with hemato-oncological malignancies, 735 (40.5%) were infected at least once with SARS-CoV-2, and 1614 (88.8%) received one or more doses of the approved vaccinations. Patients receiving antineoplastic treatment had a lower SARS-CoV-2 infection rate [35.1% versus 41.0%; odds ratio (OR) 0.78, 95% confidence interval (CI) 0.64-0.95], but higher risk of hospitalization (13.4% versus 6.9%; OR 2.11, 95% CI 1.25-3.69) compared with untreated patients. Overall, the case fatality rate (CFR) was 3.4%. Unvaccinated patients were more prone to severe coronavirus disease 2019 (COVID-19) courses requiring hospitalization (OR 2.34, 95% CI 1.25-4.36) and had a higher CFR (7.3% versus 1.6%; OR 4.98, 95% CI 2.16-12.98) than their vaccinated counterparts. In the Delta wave, patients with two vaccinations had a lower infection risk (OR 0.18, 95% CI 0.10-0.35) and tendentially lower hospitalization rates (OR 0.25, 95% CI 0.05-1.29) than unvaccinated patients. In the Omicron wave, 345/1198 (28.8%) patients with three or more vaccinations had breakthrough infections, resulting in a similar risk for infection (OR 0.88, 95% CI 0.60-1.30) but numerically lower risk for hospitalization (24/345, 7.0%) than unvaccinated individuals (4/40, 10.0%). Scheduled visits were postponed in 128/335 (38.2%) patients due to COVID-19, and deferrals correlated with pandemic wave (*P* = 0.002) and vaccination status (*P* < 0.001).

**Conclusions:**

SARS-CoV-2 infections and outcomes differ between distinct phases of the pandemic. Vaccination with variant-specific vaccines should be prioritized as general protective measures are increasingly lifted.

## Highlights

• Incidence of SARS-CoV-2 infections varied over the pandemic in patients with cancer.
• Outcomes of COVID-19 considerably differ according to pandemic phase and vaccination status.
• Overall, case fatality and hospitalization rates were lower in vaccinated patients.
• Breakthrough infections in recent waves underline the need for variant-specific vaccines.

## Introduction

Since the initial outbreak of severe acute respiratory syndrome coronavirus 2 (SARS-CoV-2) in 2019, the coronavirus disease 2019 (COVID-19) pandemic has posed significant challenges on healthcare systems in the world. Until February 2023, ∼750 million SARS-CoV-2 infections were reported worldwide, including 6.8 million fatalities.^1^ Soon it was recognized that individuals with comorbidities have a higher risk for severe clinical courses and death after SARS-CoV-2 infection.^2^ Here, immunocompromised individuals were shown to be at particularly high odds for complicated and potentially fatal disease. This includes patients after organ transplant as well as patients with hematological or solid malignancies, especially those undergoing systemic antineoplastic treatment.^3^, ^4^, ^5^ Based on these considerations, patients with cancer were among the first being prioritized for SARS-CoV-2 vaccinations according to recommendations of major hemato-oncological societies in the world.^6^^,^^7^ However, hemato-oncological patients were initially excluded from the large clinical trials leading to approval of several SARS-CoV-2 vaccines,^8^, ^9^, ^10^ and data on immune responses after vaccination are mainly derived from real-life cohorts.^11^, ^12^, ^13^, ^14^, ^15^ Here, most studies measured SARS-CoV-2-specific antibody levels in serum as a surrogate parameter for humoral immune responses; still, real-life outcome data are rare.^16^, ^17^, ^18^, ^19^

In initial reports up to 33% of patients with cancer experienced severe courses,^3^ but these numbers have considerably decreased subsequently,^20^ probably due to improved clinical management of COVID-19 and the availability of SARS-CoV-2 vaccines, but also less pathogenic variants of concern (VOC). Still, it is challenging to define the real-life efficacy of vaccinations in specific populations, such as patients with cancer, as data derived from large, unselected cohorts are limited. Moreover, the relative contribution of less pathogenic VOC and vaccinations to milder clinical courses is still not fully understood in hemato-oncological patients. By contrast, mild courses of COVID-19 may also lead to deferral of antineoplastic treatment and potentially worse oncological outcomes, underscoring the importance of ongoing protective measures for particularly vulnerable individuals.^20^

This cohort study aimed to describe the timely course of SARS-CoV-2 infections and vaccinations in a large real-life cohort of patients with hemato-oncological disease. Moreover, we sought to analyze the impact of VOC and vaccinations on infections, hospitalizations, and fatalities in distinct phases of the COVID-19 pandemic.

## Patients and methods

### Patient cohort

All patients with hematological diseases or solid tumors who were managed at the Hemato-Oncological Day Hospital Unit of the ‘Franz Tappeiner’ hospital in Merano/Meran, Italy, between 1 February 2020 and 15 December 2022 were included, regardless of whether they received antineoplastic treatment or not. Active anticancer treatment was defined as patients undergoing systemic treatment a maximum of 6 months before the infection (in line with national vaccination prioritization regulations). SARS-CoV-2 infections were diagnosed by rapid antigen testing and/or reverse transcriptase PCR of (naso-)pharyngeal swabs, where infection dates refer to the first positive testing result. Of note, SARS-CoV-2 testing was mandatory prior to each scheduled patient visit. Severity of COVID-19 was defined by treatment setting [home versus admission to non-intensive care unit (ICU) ward versus ICU]. Hospitalizations were only considered as such if COVID-19 was the reason for admission. Deaths and causes of death were recorded as documented in the official death registry. Deferrals of scheduled visits are defined as the date difference between the actual date of visit and the previously scheduled appointment. Data were retrieved retrospectively by chart review from electronic medical records.

Pandemic phases with regard to viral variants were defined based on Nextclade/Global Initiative for Sharing All Influenza Data (GISAID) data which aggregate reported results of SARS-CoV-2 genome sequencing.^21^^,^^22^ Dominant viral variants were determined according to a relative frequency of ≥50% in the reference region (Italy). Accordingly, the Alpha variant (B.1.1.7) was dominant from 1 February 2021 to 30 June 2021, the Delta variant (B.1.617.2) was dominant from 1 July 2021 to 31 December 2021, Omicron variants BA.1/BA.2 were dominant from 1 January 2022 to 15 June 2022, and Omicron variants BA.4/5 were dominant from 16 June 2022 to the database lock (i.e. 15 December 2022). Patients at risk in each pandemic wave were defined as all patients with a diagnosis of hemato-oncological disease before or during the respective pandemic phase.

All included patients were vaccinated according to national regulations. Administered vaccines included BNT162b2 (BioNTech/Pfizer), mRNA-1273 (Moderna), AZD1222 (AstraZeneca), Ad26.COV2.S (Janssen), NVX-CoV2373 (Novavax), and bivalent, variant-specific boosters BNT162b2 BA.1 and BNT162b2 BA.4/5 (BioNTech/Pfizer). To detect phase-specific changes dependent on vaccination status, outcomes for the Delta and Omicron waves were compared between unvaccinated patients at risk and those who have received two and three vaccinations, respectively, as all patients had been offered two doses by the beginning of the Delta wave and three doses by the beginning of the Omicron wave. In line with approval, one dose of Ad26.COV2.S was defined as equivalent to two doses of other vaccines.

All study procedures were conducted according to the Declaration of Helsinki with all applicable amendments as well as in compliance with institutional and national guidelines. The study was approved by the Institutional Ethics Review Board of the Südtiroler Sanitätsbetrieb (South Tyrolean Health Care Service, approval numbers 35/2020, 139/2021, 53/2022, 119/2022).

### Statistical analysis

Independence of categorical data was assessed using the chi-square and Fisher’s exact tests as appropriate. Distributions in metric data between groups were compared by applying Mann–Whitney *U* or Kruskal–Wallis tests. A two-sided *P* value of <0.05 was interpreted as statistically significant. Because of the hypothesis-generating study design, no correction for multiple testing was applied.^23^

Statistical analysis was carried out using GraphPad Prism 9.5.0 (La Jolla, CA, USA) and R version 4.2.1 (The R Foundation for Statistical Computing, Vienna, Austria) using the packages ggplot2, lubridate, cowplot, viridis, and tidyr.

## Results

### Patients’ characteristics

In total, 1817 patients with hemato-oncological disease managed between 1 February 2020 and 15 December 2022 were included, of whom 1179/1817 (64.9%) had solid tumors and 638/1817 (35.1%) had hematological malignancies. Most patients under active treatment received chemotherapy either alone (432/1817, 23.8%) or in combination with targeted or immunotherapy (88/1817, 4.8%), whereas 713/1817 (39.2%) received no antineoplastic therapy (best supportive care or regular follow-up visits). Further baseline characteristics are listed in Table 1.

**Table 1:** Baseline characteristics of the patients’ cohort

	Patient cohort (*N* = 1817), *n* (%)
**Age (years) on 15 December 2022 (or death), median (range)**	**70 (21-99)**
**Sex**	
Female	1005 (55.3)
Male	812 (44.7)
**Solid tumors**	**1179 (64.9)**
Breast cancer	332 (28.2)
Gastrointestinal cancer	309 (26.2)
Lung cancer	130 (11.0)
Urogenital cancers	213 (18.1)
Head and neck cancer	19 (1.6)
Sarcomas	29 (2.5)
Hepatobiliary cancer	47 (4.0)
Skin cancer	34 (2.9)
Central nervous system tumors	6 (0.5)
Neuroendocrine tumors	44 (3.7)
Cancer of unknown primary	8 (0.7)
Other	8 (0.7)
**Hematological malignancy**	**638 (35.1)**
Myeloproliferative diseases (polycythemia vera, essential thrombocytosis, myelofibrosis, chronic myeloid leukemia)	175 (27.4)
Chronic lymphatic leukemia	140 (21.9)
Lymphomas (indolent)	84 (13.2)
Lymphomas (aggressive)	69 (10.8)
Myelodysplastic syndrome	62 (9.7)
Hodgkin’s lymphoma	33 (5.2)
Multiple myeloma and amyloidosis	59 (9.2)
Acute leukemia	7 (1.1)
Other	9 (1.4)
**Timepoint of oncological diagnosis**	
Pre-pandemic	994 (54.7)
Pre-Alpha (1 February 2020 to 31 January 2021)	335 (18.4)
Alpha (B.1.1.7; 1 February 2021 to 30 June 2021)	156 (8.6)
Delta (B.1.617.2; 1 July 2021 to 31 December 2021)	126 (6.9)
Omicron (BA.1/2; 1 January 2022 to 15 June 2022)	124 (6.8)
Omicron (BA.4/5; 16 June 2022 to data cut-off)	82 (4.5)
**Ongoing treatment**^a^	
Chemotherapy	432 (23.8)
Immunotherapy (immune checkpoint inhibition)	106 (5.8)
Targeted therapy	205 (11.3)
Chemotherapy + targeted therapy	75 (4.1)
Chemotherapy + immunotherapy	13 (0.7)
Targeted therapy + immunotherapy	4 (0.2)
Antihormonal therapy	91 (5.0)
Targeted therapy + antihormonal therapy	28 (1.5)
Other	150 (8.3)
No antineoplastic treatment (follow-up/best supportive care)	713 (39.2)
**Stage (in patients with solid tumors, *n* = 1179)**	
Localized	411 (34.9)
Advanced	629 (53.4)
Not available/not applicable	139 (11.8)
**Number of vaccinations**^b^	
No vaccination	203 (11.2)
1 dose	8 (0.4)
2 doses	85 (4.7)
3 doses	1123 (61.8)
4 doses	382 (21.0)
5 doses	16 (0.8)
**Number of patients with confirmed SARS-CoV-2 infections (including infections prior to the diagnosis of hemato-oncological disease)**	**735 (40.5)**
1 infection	683 (37.5)
2 infections	50 (2.8)
3 infections	2 (0.1)
**Number of patients with SARS-CoV-2 infection after the diagnosis of hemato-oncological disease**	**691 (38.0)**
1 infection	650 (35.8)
2 infections	39 (2.1)
3 infections	2 (0.1)
**Infections after the diagnosis of hemato-oncological disease (*n* = 734)**	
First infection	680 (92.6)
Second infection	52 (7.1)
Third infection	2 (0.3)

SARS-CoV-2, severe acute respiratory syndrome coronavirus 2.

a Ongoing treatment at the time of infection or last documented treatment in patients with no documented infection.

b In line with national regulations and approval, one dose of AD26.COV2.S was considered as equal with two doses.

### SARS-CoV-2 vaccinations and infections over time

**Figure 1:**
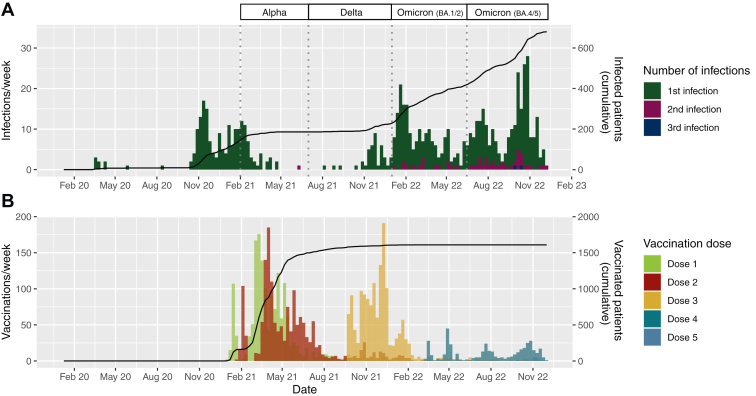
**(A) Infections and (B) vaccinations over time.** Absolute numbers per week are given in bars (left *y*-axis), cumulative numbers are given as solid line (right *y*-axis).

Infections and vaccinations over time are illustrated in Figure 1A and B, whereas used vaccines are given in Supplementary Figure S1, available at https://doi.org/10.1016/j.esmoop.2023.101559. In total (including infections prior to diagnosis of hemato-oncological disease), 735/1817 (40.5%) patients had at least one SARS-CoV-2 infection, of whom 50/1817 (2.8%) were infected two times, while a third infection was documented in 2/1817 (0.1%) patients. Notably, in 55/735 (7.5%) infected patients, the first SARS-CoV-2 infection occurred in a pandemic wave prior to hemato-oncological diagnosis; of these, 11 were infected again after the diagnosis of cancer. Overall, 1614/1817 (88.8%) patients received at least one vaccination dose. Most individuals received three doses (1123/1817, 61.8%) until data cut-off, whereas 382/1817 (21.0%) received four and 16/1817 (0.8%) received five doses.

The incidence of first SARS-CoV-2 infections after diagnosis of hemato-oncological disease was similar between patients with hematological malignancies (252/638, 39.5%) and patients with solid tumors [428/1179, 36.3%; odds ratio (OR) 0.87, 95% confidence interval (CI) 0.72-1.07; *P* = 0.179]. In the latter, less patients with advanced disease were infected (206/629, 32.8%) than patients with localized disease (179/411, 43.6%; OR 0.63, 95% CI 0.49-0.82; *P* < 0.001). Interestingly, patients under active antineoplastic treatment (388/1104, 35.1%) had a lower infection risk than those who did not receive antineoplastic treatment (292/713, 41.0%; OR 0.78, 95% CI 0.64-0.95; *P* = 0.013). However, patients receiving the B-cell-targeted agent rituximab had numerically more infections (17/37, 46.0%) than those receiving other antineoplastic agents (371/1067, 34.8%; OR 1.60, 95% CI 0.82-2.98; *P* = 0.162). Patients with verified SARS-CoV-2 infection were overall younger (median 67 years, range 21-99 years) than those who were not infected (median 71 years, range 27-97 years; *P* < 0.001).

### Disease severity in distinct phases of the pandemic

**Figure 2:**
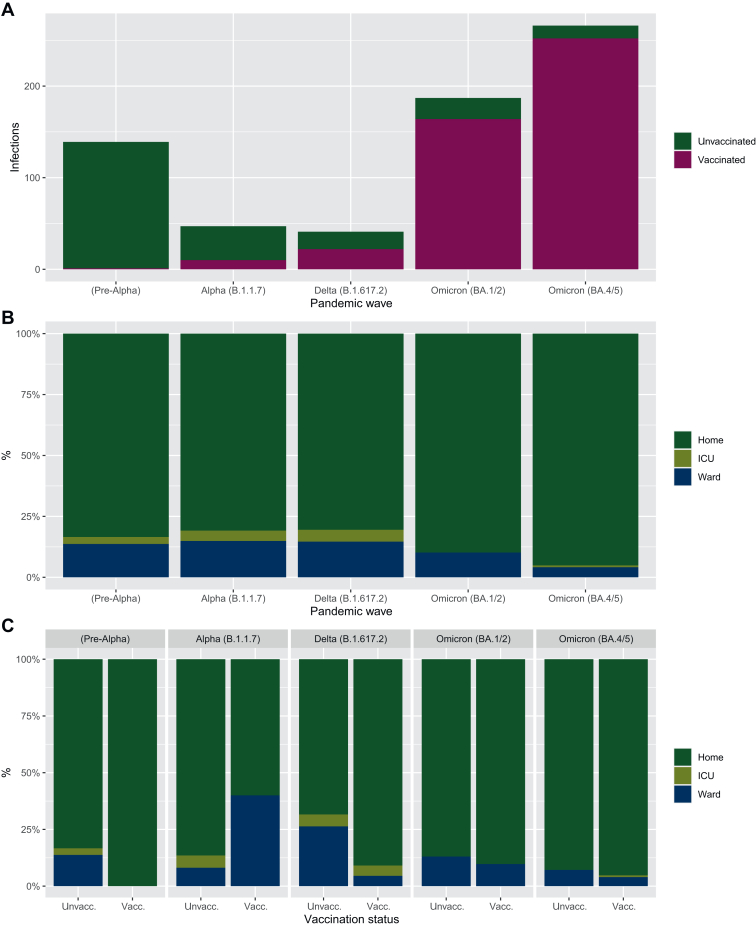
**Infections and hospitalizations in distinct phases of the pandemic.** (A) Absolute numbers of infections according to vaccination status (unvaccinated versus ≥1 vaccination), (B and C) hospitalizations and intensive care unit (ICU) admissions according to (B) pandemic phase overall and with regard to (C) vaccination status. Unvacc., unvaccinated; vacc., vaccinated.

Absolute numbers of first documented SARS-CoV-2 infections according to vaccination status (unvaccinated versus ≥1 vaccination dose) and pandemic phase by predominant viral variant (as defined in the ‘Patients and methods’ section) are given in Figure 2A, whereas disease severity (ambulant versus hospital admission versus ICU admission) in the overall cohort is shown in Figure 2B. Of note, hospitalizations (including non-ICU ward and ICU admission) after first infection were most frequently observed during the Delta wave (8/41, 19.5%), followed by Alpha (9/47, 19.1%), whereas hospital admissions were overall rare in the Omicron BA.1/2 wave (19/187, 10.2%) and the Omicron BA.4/5 wave (13/266, 4.9%; *P* < 0.001). Baseline characteristics of patients at first SARS-CoV-2 infection across pandemic phases are given in Supplementary Table S1, available at https://doi.org/10.1016/j.esmoop.2023.101559. Age, tumor entity, and applied treatment were evenly distributed. However, sex differed between pandemic phases, and infected patients with solid tumors more frequently had localized disease in the Omicron wave (135/290, 46.6%) compared with previous phases (e.g. 23/83, 27.7%, in the pre-Alpha phase in 2020).

Overall, 10 patients were admitted to the ICU over the course of the pandemic. Because of small sample sizes, further statistical analysis of ICU admissions according to pandemic wave and vaccination status was not feasible. At first infection, patients with hematological malignancies had similar hospitalization rates (28/252, 11.1%) than those with solid tumors (44/428, 10.3%; OR 1.09, 95% CI 0.67-1.81; *P* = 0.734). In patients with solid neoplasms, those with advanced disease had a higher risk for hospitalization (32/206, 15.5%) than patients with localized disease (10/179, 5.6%; OR 3.11, 95% CI 1.48-6.31; *P* = 0.002). In line with these data, infected patients undergoing active antineoplastic treatment were more likely to require hospitalization (52/388, 13.4%) than those who received best supportive care or regular follow-up visits (20/292, 6.9%; OR 2.11, 95% CI 1.25-3.69; *P* = 0.006). Similarly, patients receiving rituximab treatment were more frequently hospitalized due to COVID-19 (6/17, 35.3%) compared with other patients (46/371, 12.4%; OR 3.85, 95% CI 1.36-11.16; *P* = 0.007). At first infection, patients who required hospitalization were overall older (median age 74 years, range 40-89 years) than those who were not hospitalized (median age 66 years, range 21-99 years; *P* < 0.001).

During the observation period, 331 patients died with 25 deaths attributable to COVID-19. Of these, 24 deaths occurred after the first and one death after the second SARS-CoV-2 infection. Overall, this translates to a case fatality rate (CFR) of 3.4% (25/734 infections after diagnosis of hemato-oncological disease) and 3.5% (24/680) after first infection. Notably, the CFR after first infection was significantly lower during Omicron waves (7/453, 1.5%) as compared with previous phases of the pandemic (17/227, 7.5%; OR 5.16, 95% CI 2.24-13.45; *P* < 0.001).

### Impact of SARS-CoV-2 vaccination on infection rates and disease severity

Across pandemic phases beginning with the Alpha wave and the availability of SARS-CoV-2 vaccinations, unvaccinated patients were more likely to experience a clinical course requiring hospital admission (15/93, 16.1%) than their vaccinated counterparts (34/448, 7.6%; OR 2.34, 95% CI 1.25-4.36; *P* = 0.009; Figure 2C). This difference was particularly pronounced in the Delta wave, as 6/20 (30.0%) unvaccinated patients were hospitalized versus 2/21 (9.5%) patients who received at least one dose of an approved vaccination. Throughout the pandemic, the CFR of first infection was lower in vaccinated patients (7/448, 1.6%) than in unvaccinated patients [17/232 (7.3%); OR 4.98, 95% CI 2.16-12.98; *P* < 0.001]. Details on numbers of patients at risk and those infected in each pandemic phase are given in Supplementary Table S2, available at https://doi.org/10.1016/j.esmoop.2023.101559.

**Figure 3:**
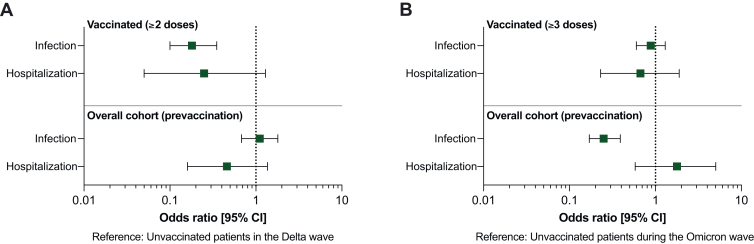
**Odds ratios for infection and hospitalization during the (A) Delta and (B) Omicron waves.** Unvaccinated patients were used as reference groups and compared with vaccinated (Delta: ≥2 doses; Omicron: ≥3 doses) patients (upper panel) and the overall cohort prior to the availability of severe acute respiratory syndrome coronavirus 2 (SARS-CoV-2) vaccines (2020, lower panel). Squares show odds ratios, whiskers represent 95% confidence intervals (CIs).

By the beginning of the Delta and the Omicron waves, all patients were offered two or three vaccinations, respectively. Therefore, to further analyze the impact of (at the time) full vaccination regimens on SARS-CoV-2 infections and disease severity, we compared unvaccinated patients with those who had received two or three vaccination doses in the Delta and Omicron waves, respectively (Figure 3A and B, Supplementary Table S2, available at https://doi.org/10.1016/j.esmoop.2023.101559). At the beginning of the Delta wave (1 July 2021), 210 patients were unvaccinated, and 1120 patients received two vaccination doses. In addition, 2 patients had received Ad26.COV2.S, resulting in 1122 patients with a vaccination status equivalent to two doses. In this pandemic phase, 21/1122 (1.9%) patients after the second vaccination dose had first SARS-CoV-2 infection compared with 20/210 (9.5%) unvaccinated patients (OR 0.18, 95% CI 0.10-0.35; *P* < 0.001; Figure 3A). Numerically, hospitalizations were less frequent in patients who had received two vaccination doses (2/21, 9.5%) compared with their unvaccinated counterparts (6/20, 30.0%; OR 0.25, 95% CI 0.05-1.29; *P* = 0.130). Moreover, infection rates were similar in 2020 before general availability of SARS-CoV-2 vaccines (139/1329, 10.5%) compared with unvaccinated patients during the Delta wave (20/210, 9.5%; OR 1.11, 95% CI 0.68-1.80; *P* = 0.679). Furthermore, there was a numerical trend toward lower hospitalization rates in the prevaccination era (23/139, 16.6%) compared with unvaccinated patients during the Delta wave (6/20, 30.0%; OR 0.46, 95% CI 0.16-1.36; *P* = 0.145), suggesting similar transmissibility but higher pathogenicity of the Delta VOC compared with earlier phases of the pandemic.

At the beginning of the Omicron wave (1 January 2022), 1137 patients had received three vaccination doses, while 123 remained unvaccinated. In total, 345/1198 (28.8%) patients after third vaccination were infected compared with 40/127 (31.5%) unvaccinated patients (OR 0.88, 95% CI 0.60-1.30; *P* = 0.524; Figure 3B), indicating a considerable number of breakthrough infections. However, hospitalizations after first infection were numerically less frequent in patients who had received three vaccination doses (24/345, 7.0%) versus unvaccinated patients (4/40, 10.0%; OR 0.67, 95% CI 0.23-1.89; *P* = 0.483). Patients in the pre-Alpha phase prior to general availability of SARS-CoV-2 vaccines were less likely to be infected (139/1329, 10.5%) than unvaccinated patients during the Omicron wave (40/127, 31.5%; OR 0.25, 95% CI 0.17-0.39; *P* < 0.001). However, no differences were seen with regard to hospitalization [prevaccination: 23/139 (16.6%) versus unvaccinated Omicron: 4/40 (10.0%); OR 1.78, 95% CI 0.58-5.03; *P* = 0.308].

### Deferral of scheduled visits and antineoplastic treatment

Of all 691 patients who were infected at least once with SARS-CoV-2 after diagnosis of hemato-oncologic disease, scheduled visits including administration of antineoplastic treatment were postponed in 335/691 (48.5%) cases, of which 128/335 (38.2%) deferrals were related to SARS-CoV-2 infections.

**Figure 4:**
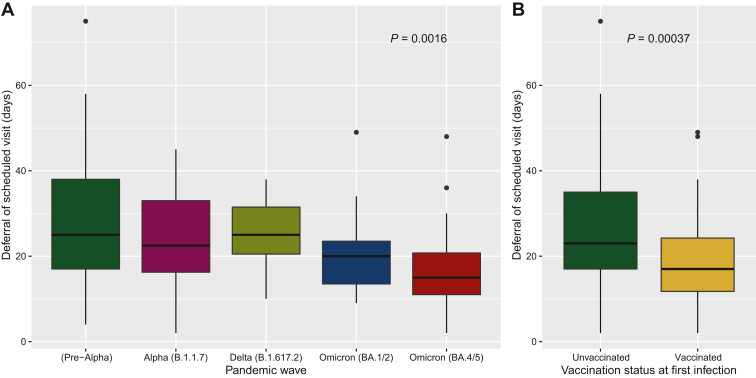
**Length of deferrals of scheduled visits according to (A) pandemic wave and (B) vaccination status.** P values as determined by the Kruskal–Wallis test (A) and Mann–Whitney *U* test (B).

In median, deferrals due to COVID-19 had a duration of 20 days (range 2-75 days) and differed between pandemic waves (*P* = 0.002; Figure 4A), with the longest deferrals in 2020 (median 25 days, range 4-75 days) and during the Delta wave (median 25 days, range 10-38 days), while scheduled follow-up visits were postponed for only a median of 15 days during the Omicron BA.4/5 phase (range 2-48 days). Moreover, delays were shorter in vaccinated (median 17 days, range 2-49 days) compared with unvaccinated patients (median: 23 days, range 2-75 days; *P* < 0.001; Figure 4B) throughout the pandemic.

## Discussion

Herein, we analyzed the time course of SARS-CoV-2 infections in a cohort of patients with hemato-oncological diseases, reflecting real-life outcomes of COVID-19 in an unselected patient population throughout the pandemic. Overall, ∼40% of patients were infected at least once over the observation period, and ∼90% in our cohort received at least one vaccination dose. By contrast, ∼55% of the overall regional population was infected,^24^ and ∼78% of those eligible for vaccination received at least one dose until 23 December 2022.^25^ These numbers underscore the high awareness of oncological patients and caregivers for COVID-19 and protective measures including vaccination. Indeed, our cohort also presented with a higher rate of hospitalization and fatalities than in the overall population, supporting previous reports of more complicated clinical courses in patients with cancer, especially those undergoing systemic antineoplastic treatment.^3^^,^^20^^,^^26^, ^27^, ^28^ In line with data on attenuated humoral immune responses after vaccination and previous clinical data,^26^^,^^29^, ^30^, ^31^ patients receiving the B-cell-targeting agent rituximab were particularly prone to SARS-CoV-2 infections and hospitalization in our cohort.

Because of the long observation period from February 2020 to December 2022, our study allowed to gain further insights into the impact of distinct pandemic phases on infections and their outcomes while local protective measures were kept continuously maintained. Indeed, infections, hospitalizations, and deaths due to COVID-19 differed considerably between pandemic waves in both the general population and in patients with cancer as previously reported.^32^, ^33^, ^34^ However, increased infection rates and a lower risk for hospitalization for Omicron variants probably result from a combination of virus- and host-related factors including increased transmissibility and reduced pathogenicity, as well as enhanced immunity in the population by both vaccination and previous infections. Indeed, while vaccination remains a protective factor against severe COVID-19 in patients infected with the Omicron variant, previous data also suggest reduced hospital admissions in unvaccinated patients compared with the Delta VOC.^35^^,^^36^ As still ∼12% of patients in our cohort were unvaccinated, comparisons according to vaccination status could be made in distinct phases of the pandemic. Indeed, we could find a tendentially higher risk for hospitalization in unvaccinated patients during the Delta wave compared with vaccinated individuals and the prevaccination period, suggesting both a real-life efficacy of vaccination and a higher pathogenicity of the Delta VOC compared with pre-Alpha variants. We could also observe high rates of breakthrough infections during the Omicron waves, emphasizing the immune-evading abilities of these VOCs. While lockdowns were increasingly lifted for vaccinated individuals, mobility restrictions remained in place for the unvaccinated population,^37^ probably contributing to relatively high infection rates in vaccinated patients, although hospitalization rates and fatalities remained low.

In the first days of the pandemic and in light of shortages in healthcare resources, disruption in cancer care has been discussed and specific subgroups such as patients receiving adjuvant treatment have been prioritized for treatment continuation.^38^ Later on, reasons for treatment deferrals increasingly shifted toward individual SARS-CoV-2 infections and postinfection sequelae, with significant impact on cancer-related survival.^20^ In our cohort, more than one-third of treatment disruptions were due to COVID-19 with a median duration of almost 3 weeks, potentially affecting outcomes of oncological treatments. Whereas previous reports showed decreased severity for Omicron BA.1/BA.2 variants also in patients with cancer,^16^^,^^39^^,^^40^ our data suggest even milder courses for Omicron BA.4/5, although increased vaccination coverage and the increasing application of bivalent, variant-specific vaccines may have contributed. However, infections still inevitably lead to delays in anticancer treatment also in the case of mild clinical courses, highlighting the urgent need for continuous vaccination efforts with VOC-specific boosters especially in vulnerable patient populations as currently discussed.^41^

Clearly, this study has limitations which mainly lie in its retrospective design in an unselected patient cohort, leading to inherent heterogeneity of the population, small sample sizes in certain subgroups, and missing information, particularly regarding performance status as this may represent a further confounder impacting outcomes after SARS-CoV-2 infection. Moreover, we did not consider disease stage in hematological malignancies as these include very heterogeneous entities with no uniform staging system. Furthermore, the low number of patients, infections, and hospitalizations in certain subgroups precluded multivariate analysis to correct for confounding factors. Whereas data on vaccinations and infections have been systematically selected and patients underwent regular SARS-CoV-2 testing prior to scheduled visits, asymptomatic and undetected infections between visits cannot be excluded, especially in patients who had follow-up visits in larger intervals as compared with those undergoing active antineoplastic treatment. In addition, data on further SARS-CoV-2 medication such as antiviral agents including nirmatrelvir/ritonavir or molnupiravir have not been recorded. Lastly, the number of ICU admissions was relatively low; here, triage decisions in earlier phases of the pandemic with shortage of ICU resources may have contributed. This precluded further distinction to non-ICU wards when considering disease severity.

In conclusion, our study provides a bird’s eye view on infection and vaccination dynamics throughout the pandemic, considering the impact of distinct viral variants and SARS-CoV-2 vaccinations, which both have contributed to vastly differing infection numbers and disease severity. Although the frequency of severe courses is decreasing, vaccination of patients with cancer with variant-specific boosters should be further supported as antineoplastic treatments are still postponed due to COVID-19, potentially leading to adverse oncological outcomes.
