# Supplementary figures and images for: Timely course of SARS-CoV-2 infections and vaccinations in patients with hemato-oncological diseases: analysis of a real-life cohort
Source: ESMO Open. 2023 Apr 25;8(3):101559. doi: 10.1016/j.esmoop.2023.101559 (PMC10126224; doi:10.1016/j.esmoop.2023.101559)

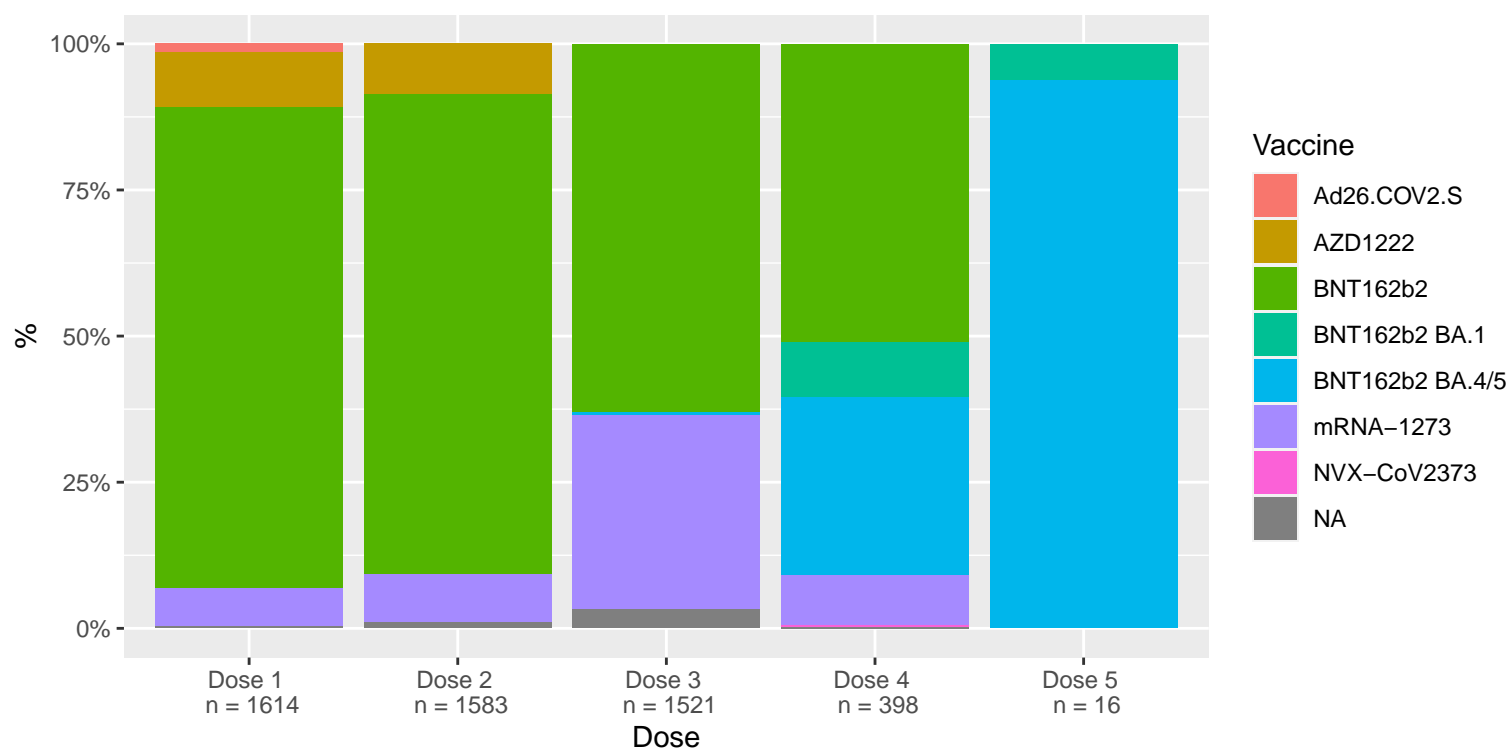

Supplement: Supplementary Figure S1 [file mmc1.pdf]
