# Supplementary material for: Timely course of SARS-CoV-2 infections and vaccinations in patients with hemato-oncological diseases: analysis of a real-life cohort
Source: ESMO Open. 2023 Apr 25;8(3):101559. doi: 10.1016/j.esmoop.2023.101559 (PMC10126224; doi:10.1016/j.esmoop.2023.101559)
Supplement: Supplementary Tables [file mmc3.docx]

**Supplementary Material**

**Supplementary Figure 1. Used vaccines for first to fifth vaccination dose.** AD26.COV2.S = Janssen; AZD1222 = AstraZeneca; BNT162b2 = BioNTech/Pfizer (including bivalent, variant specific BNT162b2 BA.1 and BNT162b2 BA.4.5); mRNA-1273 = Moderna; NVX-CoV2373 = Novavax.

**Supplementary Table 1. Baseline characteristics of infected patients in distinct phases of the pandemic (at first infection).** P-values as determined by Chi-square/Fisher’s exact and Kruskal-Wallis tests as appropriate.

**Supplementary Table 2. Numbers of patients at risk, infections and hospitalizations in distinct phases of the pandemic.**
